# Synergistic Cytotoxicity of a Toxin Targeting the Epidermal Growth Factor Receptor and the Glycosylated Triterpenoid SO1861 in Prostate Cancer

**DOI:** 10.7150/jca.85691

**Published:** 2023-09-18

**Authors:** Alexandra Fischer, Anie Priscilla Masilamani, Susanne Schultze-Seemann, Isis Wolf, Christian Gratzke, Hendrik Fuchs, Philipp Wolf

**Affiliations:** 1 Department of Urology, Medical Center—University of Freiburg, Freiburg, Germany.; 2 Faculty of Medicine, University of Freiburg, Freiburg, Germany.; 3 Faculty for Biology, University of Freiburg, Freiburg, Germany.; 4 Charité - Universitätsmedizin Berlin, corporate member of Freie Universität Berlin and Humboldt-Universität zu Berlin; Institute of Diagnostic Laboratory Medicine, Clinical Chemistry and Pathobiochemistry; Berlin, Germany.

**Keywords:** EGF, EGFR, targeted toxin, endosomal escape enhancer, SO1861, prostate cancer

## Abstract

Treatment of advanced prostate cancer lacks specificity and curative intent. Therefore, the need for new targeted therapeutic approaches is high. In the present study, we generated the new targeted toxin EGF-PE24mutΔREDLK binding to the epidermal growth factor receptor (EGFR) on the surface of prostate cancer cells. It consists of the human epidermal growth factor (EGF) as binding domain and a de-immunized variant of *Pseudomonas* Exotoxin A (PE), called PE24mutΔREDLK, as toxin domain. The toxin domain contains a deletion of the C-terminal KDEL-like motif REDLK to prevent its transport from sorting endosomes via the KDEL receptor mediated pathway into the cytosol, where it can inhibit cellular protein biosynthesis and induce apoptosis. Indeed, REDLK deletion resulted in a strong decrease in cytotoxicity of the targeted toxin in prostate cancer cells compared to the parental targeted toxin EGF-PE24mut. However, addition of the plant glycosylated triterpenoid SO1861, which is known to mediate the release of biomolecules from endolysosomal compartments into the cytosol, resulted in an up to almost 7,000-fold enhanced synergistic cytotoxicity. Moreover, combination of PE24mutΔREDLK with SO1861 led to a cytotoxicity that was even 16- to 300-fold enhanced compared to that of EGF-PE24mut. Endolysosomal entrapment of the non-toxic targeted toxin EGF-PE24mutΔREDLK followed by activation through enhanced endosomal escape therefore represents a new promising approach for the future treatment of advanced prostate cancer with high efficacy and diminished side effects.

## Introduction

In recent decades, diagnosis of prostate cancer has improved and treatment of early tumor stages has progressed, e.g. by robot-assisted surgery 1. Despite new combinatorial therapies comprising hormone deprivation, chemotherapy, radiation and immunotherapy, however, this tumor entity remains a deadly disease in advanced, metastatic stages 2. In the increasing context of personalized cancer therapy, targeted approaches come more and more into focus that include the use of radioligands, antibody-drug conjugates, vaccines, immune checkpoint inhibitors, CAR T cells and bispecific T-cell engagers 3-6.

Another field of interest adresses the development of targeted toxins, recombinant proteins with two main domains: a binding domain (e.g., antibody, antibody fragment or ligand), which specifically binds to a target antigen on the surface of cancer cells, and a toxin domain of either bacterial or plant origin that unfolds its cytotoxic effect after cellular internalization 7. Previously, various targeted toxins have been developed preclinically for the treatment of prostate cancer. They comprise variants with antibodies or antibody fragments against CD44, Her-2, fibroblast growth factor receptor (FGFR) or the prostate specific membrane antigen (PSMA) as binding domains and saporin from the plant *Saponaria officinalis* or the toxic part of *Pseudomonas* Exotoxin A (PE) from the bacterium *Pseudomonas aeruginosa* as toxin domains 8.

Recently, we generated targeted toxins binding to the epidermal growth factor receptor (EGFR) for the treatment of prostate cancer 9. EGFR signaling pathways promote proliferation, survival, invasiveness and metastasis of prostate cancer cells 10-12 and EGFR expression correlates with grading, recurrence, poorer metastasis-free survival and therapeutic resistance of prostate cancer 13-16. Our targeted toxins EGF-PE40 and EGF-PE24mut were generated by fusion of the human EGF ligand to the natural toxic domain of PE with 40 kDa in size, called PE40, or to a de-immunized variant thereof with 24 kDa in size, called PE24mut. EGF-PE40 and EGF-PE24mut were able to specifically kill EGFR-expressing prostate cancer cells and were found to be more than 60- to 140,000-fold cytotoxic than the EGFR inhibitor erlotinib 9.

The last amino acids at the C-terminus (REDLK) of the toxic PE40 or PE24mut domains form a KDEL-like motif. After internalization of targeted toxins into early endosomes and enzymatic cleavage of the toxic domain by the subtilisin-like proprotein convertase furin, it can bind to KDEL receptors in the Golgi apparatus and travels retrogradely to the endoplasmic reticulum (ER) 17. In this manner, the toxin exploits a pathway by which cellular proteins with KDEL sequences return to the ER after accidental export, and which helps to maintain the homeostasis of the cellular transport apparatus 18. Once the toxic domain of PE is secreted from the ER into the cytosol, it specifically ADP-ribosylates a modified histidine residue, called diphthamide, of the eukaryotic elongation factor-2 (eEF-2), which promotes the GTP-dependent translocation of the ribosome. ADP-ribosylation of eEF-2 results in an overall protein biosynthesis inhibition and apoptosis of the intoxicated cells 17.

Intracellular trafficking of the toxic domain of PE from the endosomes into the cytosol does not proceed without loss. After internalization, it can be transported to lysosomes and degraded before it travels to the cytosol 17. This implies that higher doses of targeted toxins are required to effectively kill the target cells than necessary. In a clinical setting, this might result in dose limitations due to enhanced side effects. Therefore, new strategies are being developed to protect PE-based targeted toxins from lysosomal degradation or to augment their cytosolic release, including cell-penetrating or fusogenic peptides and light-induced techniques 19-21.

The aim of the present study was to optimize the efficacy of the targeted toxin EGF-PE24mut for the treatment of prostate cancer. For this purpose, we deleted the C-terminal KDEL-like amino acid sequence REDLK for endolysosomal entrapment and added the glycosylated triterpenoid SO1861 from *Saponaria officinalis,* which has the ability to mediate the transport of proteins, peptides, DNA and siRNA directly through the endolysosomal membrane into the cytosol 22-25. Combination of EGF-PE24mutΔREDLK with SO1861 resulted in synergistic cytotoxic effects in prostate cancer cells and was even more effective than the targeted toxin containing the PE24mut domain. The combination therapy therefore represents a new promising therapeutic option for the future treatment of advanced prostate cancer.

## Materials and Methods

### Cell culture and chemicals

The prostate cancer cell lines LNCaP, DU145 and PC-3 were purchased from the American Type Culture Collection (ATCC, Manassas, VA, USA). All cells were shown to express EGFR in one of our earlier publications 9. The EGFR-negative control cell line CHO was obtained from Gibco (Karlsruhe, Germany). LNCaP and DU145 cell were cultured in RPMI 1640 medium (Thermo Fisher Scientific, Schwerte, Germany) and PC-3 and CHO cells in F-12 Nut Mix medium (Life Technologies), all supplemented with 10% FCS (Biochrom, Berlin, Germany) + 1% penicillin/streptomycin (Biochrom) and grown at 37°C and 5% CO_2_ in a humid atmosphere. The *pan-*caspase inhibitor QVD-OPh hydrate (3S)-5-(2,6-difluorophenoxy)-3-[[(2S)-3-methyl-2-(quinoline-2-carbonylamino)butanoyl]amino]-4-oxopentanoic acid) was purchased from Sigma-Aldrich (Taufkirchen, Germany) and stored as a 25 mM solution in DMSO at 4˚C.

### Generation of the targeted toxins EGF-PE24mut and EGF-PE24mutΔREDLK

**Figure 1:**
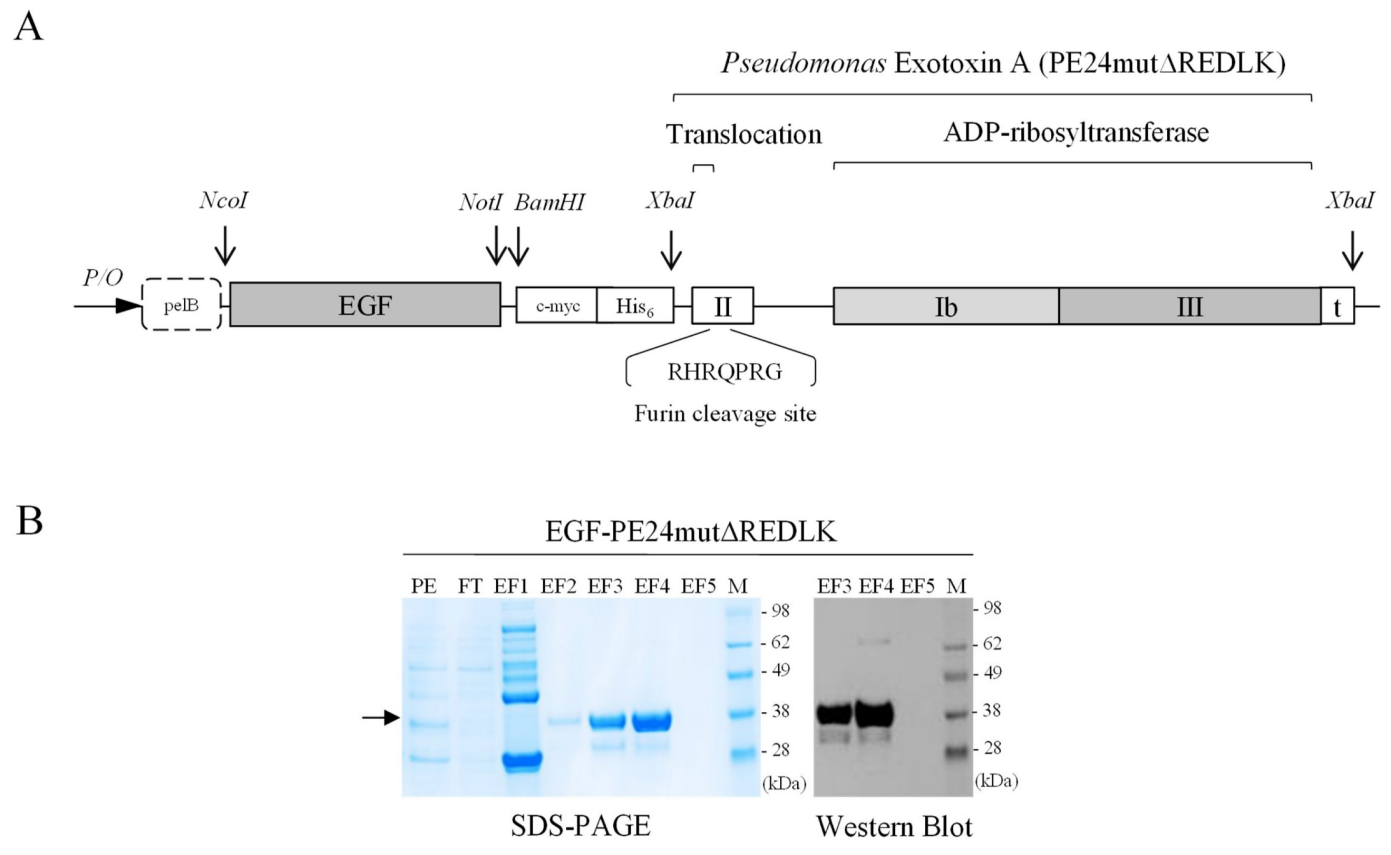
** Generation of the targeted toxin EGF-PE24mutΔREDLK. (A)** Schematic representation of the targeted toxin in the expression vector pHOG21. **(B)** SDS-PAGE and Western Blot of EGF-PE24mutΔREDLK. The targeted toxin was found in the elution fractions EF3 and EF4 after purification by IMAC (arrow). Abbreviations: PE, periplasmatic extract; FT, flow through; EF, elution fraction, M, protein marker.

The targeted toxin PE24mut was cloned, expressed and purified as described previously 9. The targeted toxin EGF-PE24mutΔREDLK, lacking the C-terminal REDLK sequence, was generated as follows. The gene of the toxin domain PE24mutΔREDLK including a stop codon was codon optimized for expression in *E.coli*, synthesized (Geneart, Regensburg, Germany) and cloned via the *XbaI* restriction site into the expression vector pHOG21 C-terminally to the sequence of the human EGF ligand (Fig. 1A). The pHOG21 vector contains a *pelB* leader sequence for periplasmatic expression and a human *c-myc* tag and a *hexahistidine* tag for detection and purification, respectively. EGF-PE24mutΔREDLK was periplasmatically expressed in *E.coli* bacteria and purified by immobilized affinity chromatography (IMAC) using HiTrap^TM^ Chelating High Performance columns (Sigma-Aldrich, St. Louis, MO, USA) as described 9. The targeted toxin was stepwise eluted from the columns by increasing imidazole concentrations (40 mM - 250 mM) and dialyzed against PBS. Protein concentration of the purified targeted toxin was determined by NanoDrop^TM^ Lite Spectrophotometer (Thermo Fisher Scientific, Waltham, MA, USA).

### Generation of the plant glycosylated triterpenoid SO1861

SO1861 was isolated from *Saponaria officinalis* L. as described 26. Purity and identity were analyzed by LC/MS with an Agilent 6210 TOF LC/MS system. SO1861 was dissolved at a concentration of 200 μg/ml in distilled water, aliquoted and stored at -20°C. After thawing, SO1861 was incubated at 50 °C for 10 min and homogenized in an ultrasonic bath before use.

### SDS-PAGE and Western Blot analyses

The targeted toxin EGF-PE24mutΔREDLK was detected by SDS-PAGE and Western Blot analysis in the elution fractions after purification using the horseradish peroxidase (HRP)-conjugated mouse anti human c-myc mAb (Cat. No. 11814150001, Roche Diagnostics, Mannheim, Germany) as detection antibody.

For the analysis of protein biosynthesis inhibition, target cells were treated with EGF-PE24mutΔREDLK and SO1861 for 48 or 72 h, respectively. Then they were incubated with 5 µg/ml puromycin (Tocris, Bio-Techne GmbH, Wiesbaden, Germany) for 15 min and lysed in 50 mM Tris-HCl, 150 mM NaCl, 1 mM EDTA, 0.5% NaDeoxycholate, 0.05% SDS, 1% Igepal. Mouse anti-puromycin mAb (Cat. No. MABE343, Merck, Darmstadt, Germany) and HRP-conjugated rabbit anti-mouse Ab (Cat. No. P0161, Dako, Hamburg, Germany,) were used for the detection of proteins by Western Blot that were translated at the time of lysate preparation. β-actin was detected as a loading control by HRP-labeled rabbit mAb (Cat. No. 4970, Cell Signaling Technology Europe, Leiden, The Netherlands).

Induction of apoptosis was proven in Western Blot analysis by detection of poly (ADP-ribose) polymerase (PARP) cleavage using the antibodies PARP rabbit pAb (Cat. No. 9542, Cell Signaling Technology Europe,) and HRP conjugated goat anti-rabbit pAb (Cat. No. P0448, Dako, Hamburg, Germany) and by detection of caspase-3 activation using the Cas-3 mouse mAb (Cat. No. CM4911, ECM Biosciences, Versailles, KY, USA) and HRP-conjugated rabbit anti-mouse Ab (Cat. No. P0161, Dako). Western blots were developed with an enhanced chemiluminescence (ECL) system and protein bands were detected and analyzed with the help of a ChemoDoc^™^ MP Imaging System and the software Image Lab™ (Bio-Rad Laboratories, Feldkirchen, Germany).

### Flow Cytometry

Binding of EGF-PE24mut and EGF-PE24mutΔREDLK to the prostate cancer cells was analyzed by flow cytometry as described 27. Rabbit anti-human His tag mAb (Cat. No. 12698, Cell Signaling Technology Europe, Leiden, The Netherlands) and PE conjugated goat anti-rabbit IgG (Cat. No. 4010-09S, Southern Biotech, Birmingham, AL, USA) were used for the detection of bound targeted toxins.

### Cell viability assays

Cell viability after treatment with the targeted toxins and SO1861 was measured using 4-[3-(4-iodophenyl)-2-(4-nitrophenyl)-2H-5-tetrazolio]-1,3-benzene disulfonate - water-soluble tetrazolium 1 (WST-1) - assay according to the manufacturer's instructions. In brief, 1.5×10^4^ cells / well were seeded into 96 well plates and incubated overnight at 37°C and 5% CO_2_ for adherence. Then the cells were treated with SO1861 for 15 min followed by incubation with the targeted toxins for 48 h (LNCaP cells) or 72 h (DU145, PC-3 and CHO cells). Finally, WST-1 reagent was added, and plates were incubated until the maximum absorbance at 450 nm in the untreated control wells reached values of about 2.5 optical densities (OD). Quantification of apoptosis and necrosis after treatment was analyzed using the Apoptosis and Necrosis Quantification Kit Plus in accordance with the manufacturer's protocol (Biotium, Fremont, CA, USA).

### Phase-contrast microscopy

Morphology of the prostate cancer cells after treatment was analyzed with the help of the Zeiss AxioObserver Z.1 inverted microscope and the softwares ZEN 2 Pro und ZEN 2.0 (Carl Zeiss Microscopy GmbH, Munich, Germany).

### Statistics

Equilibrium dissociation constants (Kd), defined as the ratio of the dissociation rate to the association rate - and corresponding to targeted toxin concentrations leading to half-maximal antigen binding saturation, if a 1:1 complex is formed - were determined using the flow cytometric data and the software GraphPad Prism 8.01. The IC_50_ values, defined as the targeted toxin concentrations leading to a 50% reduction in cell viability compared to untreated control cells, as measured by WST-1 assay, were calculated by non-linear regression [log (inhibitor) vs. response (three parameters)] using the same software. The cytotoxicity of EGF-PE24mutΔREDLK in combination with SO1861 was characterized as synergistic or antagonistic for each tested toxin concentration using the *Bliss Independence* Model 28, 29. According to this model, the treatment effect *E* for each tested drug concentration is defined as the percentage of viable tumor cells that was reduced due to therapy (inhibition rate). The predicted inhibition rate (*Êa+b*) of the combination therapy can be calculated using the following equation:

*Ê_a+b_* = *E_a_* + *E_b_* - (E_a_*E_b_); 0 ≤ *E_i_* ≤ 1; *i* = *a*, *b*, *a* + *b*;

with a = EGF-PE24mut-ΔREDLK, b = SO1861 and a + b = combination.

E_a_ and E_b_ are the observed inhibition rates with EGF-PE24mut-ΔREDLK or SO1861 alone. The difference between the measured inhibition rate (*Ea+b*) and the predicted inhibition rate (*Êa+b*) corresponds to the *Bliss Independence* score, whereby values > 0 indicate synergism and values < 0 indicate antagonism. Statistical significance values (p-values) were calculated on the untreated control or the individual treatments in GraphPad Prism 8.01 using the unpaired *t-*test with Welch's correction.

## Results

The targeted toxin EGF-PE24mutΔREDLK was successfully expressed in the periplasm of *E. coli* bacteria and purified by IMAC. EGF-PE24mutΔREDLK was detected by SDS-PAGE in the elution fractions EF3 and EF4 in high purity. Western Blot analysis confirmed the expression of the 34.1 kDa protein (Fig. 1B). Binding of EGF-PE24mut and EGF-PE24mutΔREDLK to prostate cancer cells was determined by flow cytometry. K_d_ values between 26.6 and 36.9 nM for EGF-PE24mut and between 23.3 and 32.3 nM for EGF-PE24mutΔREDLK were calculated (Table 1). This proved that the deletion of the REDLK sequence did not impair the binding affinity of the targeted toxin to EGFR. No binding was found to EGFR-negative CHO cells, which demonstrated the high specificity of the targeted toxins.

**Figure 2:**
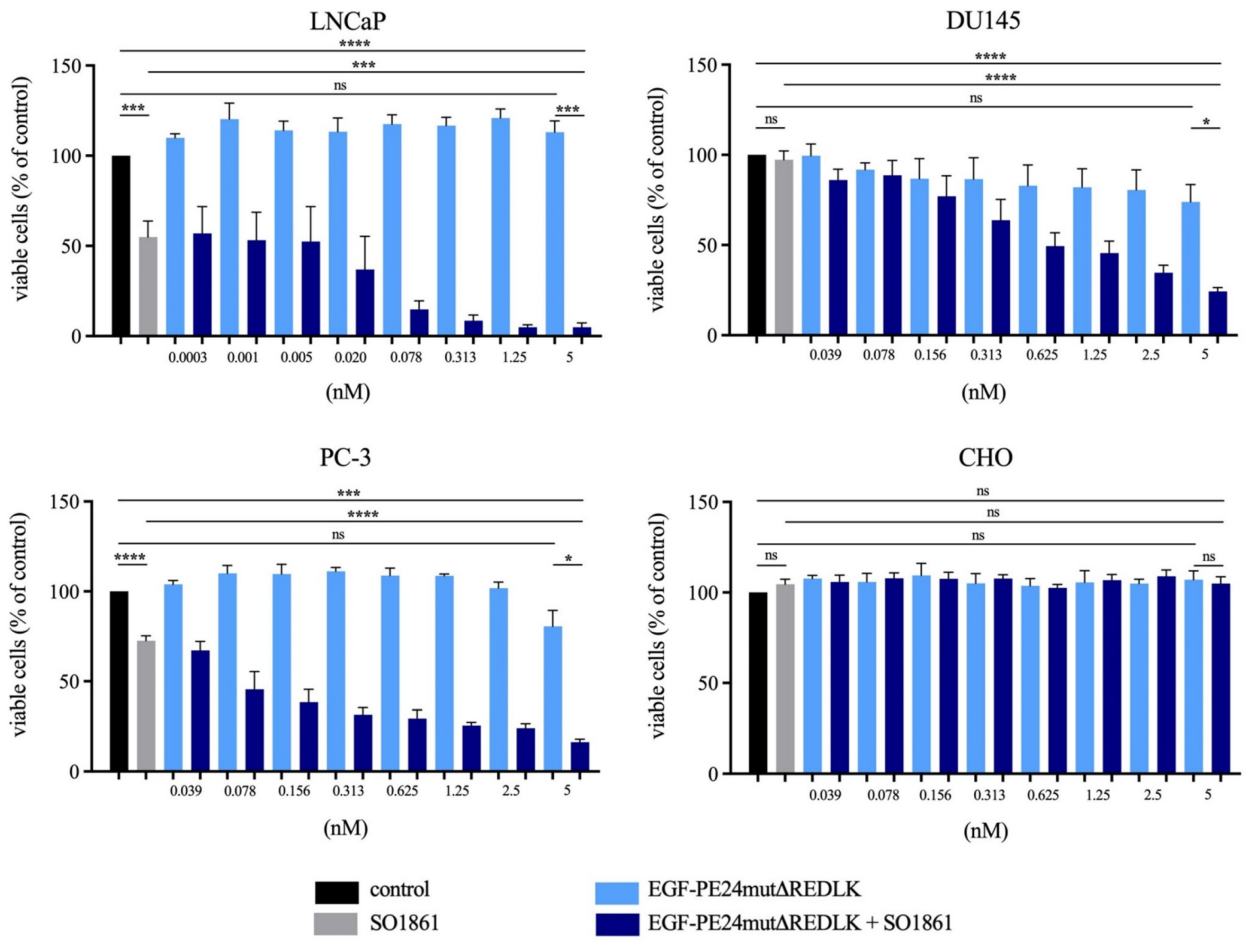
** Cytotoxicity of EGF-PE24mutΔREDLK in combination with SO1861 in prostate cancer cells. (A)** Cell viability was determined by WST-1 assay after 48 h (LNCaP) or 72 h incubation (DU145, PC-3, CHO) with EGF-PE24mutΔREDLK alone or in combination with a subtoxic concentration of 1 µg/ml SO1861. Mean values +/- SEM of three independent experiments. Statistical significance was calculated by unpaired *t-*test with Welch's correction with ns, not significant; ***, p < 0.001; ****, p < 0.0001.

EGF-PE24mut showed high and specific cytotoxicity in the EGFR-expressing prostate cancer cells. With an IC_50_ value of 0.9 nM after 48 h incubation, LNCaP cells were found to be most sensitive to the targeted toxin. For DU145 and PC-3 cells, IC_50_ values of 15.8 nM and 1.6 nM were determined after 72 h incubation. In contrast, cytotoxicity of EGF-PE24mutΔREDLK was substantially reduced and IC_50_ values were not reached for LNCaP, DU145 and PC-3 cells within a concentration range of up to 20.9 nM (Table 2). Moreover, both targeted toxins were not cytotoxic in EGFR-negative CHO cells. Addition of 1 µg/ml of the endosomal escape enhancer SO1861 led to a 4- to 150-fold enhanced cytotoxicity of EGF-PE24mut (Table 2) and even to a more than 26.2- to 6,966-fold enhanced cytotoxicity of EGF-PE24mutΔREDLK in the prostate cancer cell lines (Table 2, Fig. 2A).

Implementing the *Bliss Independence* score revealed that the addition of 1 µg/ml SO1861 led to synergistic cytotoxic effects at targeted toxin concentrations between 0.001 and 5 nM in LNCaP cells (after 48 h), between 1.25 and 5 nM in DU145 cells and between 0.039 and 5 nM in PC-3 cells (both after 72 h), identifying an optimal range of non-toxic EGF-PE24mut-ΔREDLK concentrations for each cell line that induced a significant cytotoxicity, when simultaneously applied with SO1861 (Table 3). In all prostate cancer cell lines, the cytotoxic effect of EGF-PE24mut-ΔREDLK was not only reconstituted, but also multiplied by endosomal escape enhancement, despite its at least 11-fold lower intrinsic toxicity compared to EGF-PE24mut.

**Figure 3:**
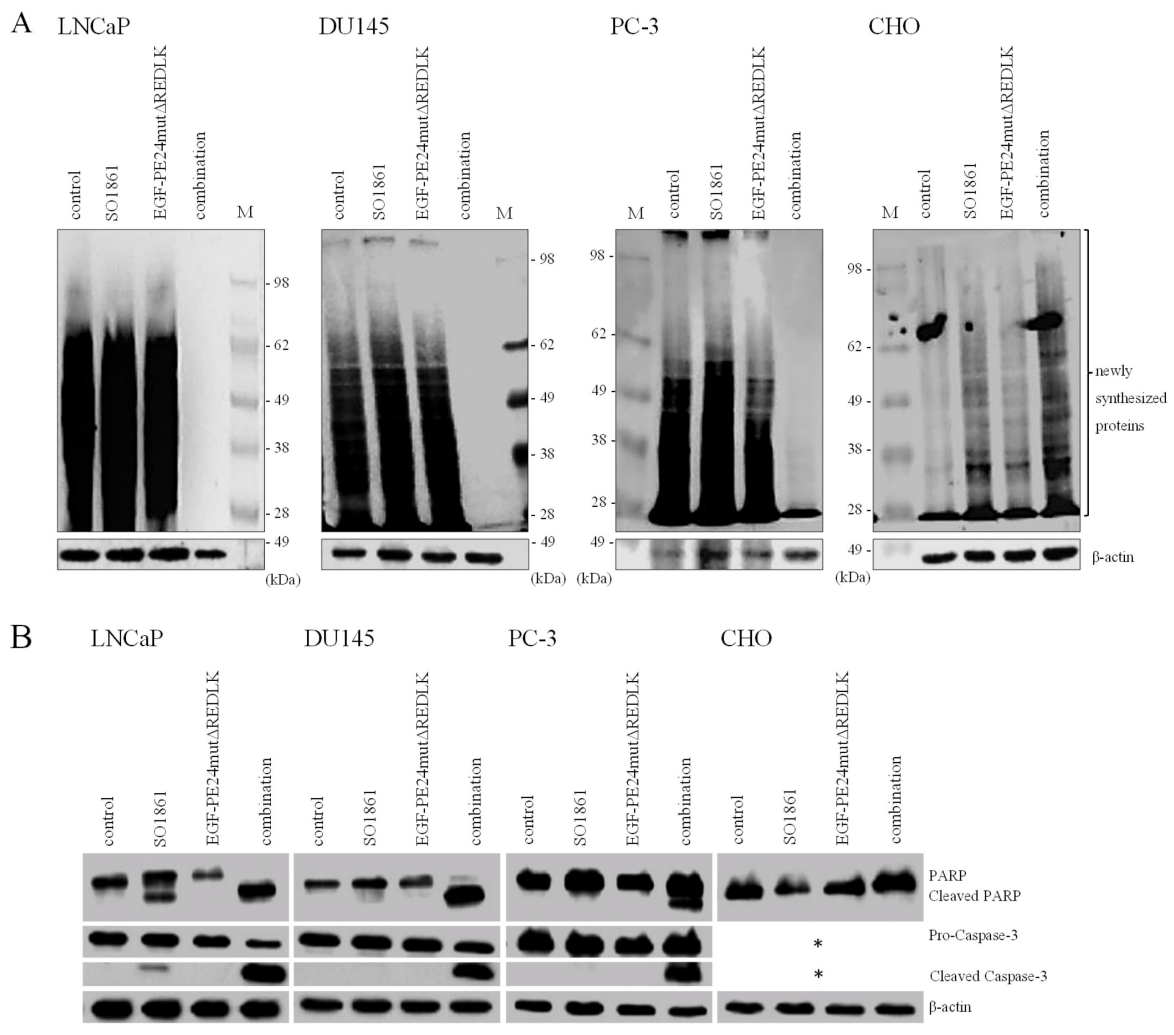
** Inhibition of protein biosynthesis and induction of apoptosis in prostate cancer cells after treatment with EGF-PE24mutΔREDLK and SO1861. (A)** Protein biosynthesis inhibition was demonstrated by puromycin Western Blot after 48 h in LNCaP and after 72 h in DU145 and PC-3 cells. EGFR-negative CHO cells remained unaffected. **(B)** Induction of apoptosis after combination treatment was marked by poly (ADP-ribose) polymerase (PARP) cleavage and caspase-3 activation. Western blots after 48 or 72 h of single or combination treatment of LNCaP (48 h), DU145, PC-3 or CHO cells (all 72 h) with 1.0 µg/ml SO1861 and 2.5 nM EGF-PE24mutΔREDLK. β-actin was used as a loading control. * As CHO cells are not of human origin, detection of human Caspase-3 was not possible in this cell line. Abbreviation: M, protein marker.

**Figure 4:**
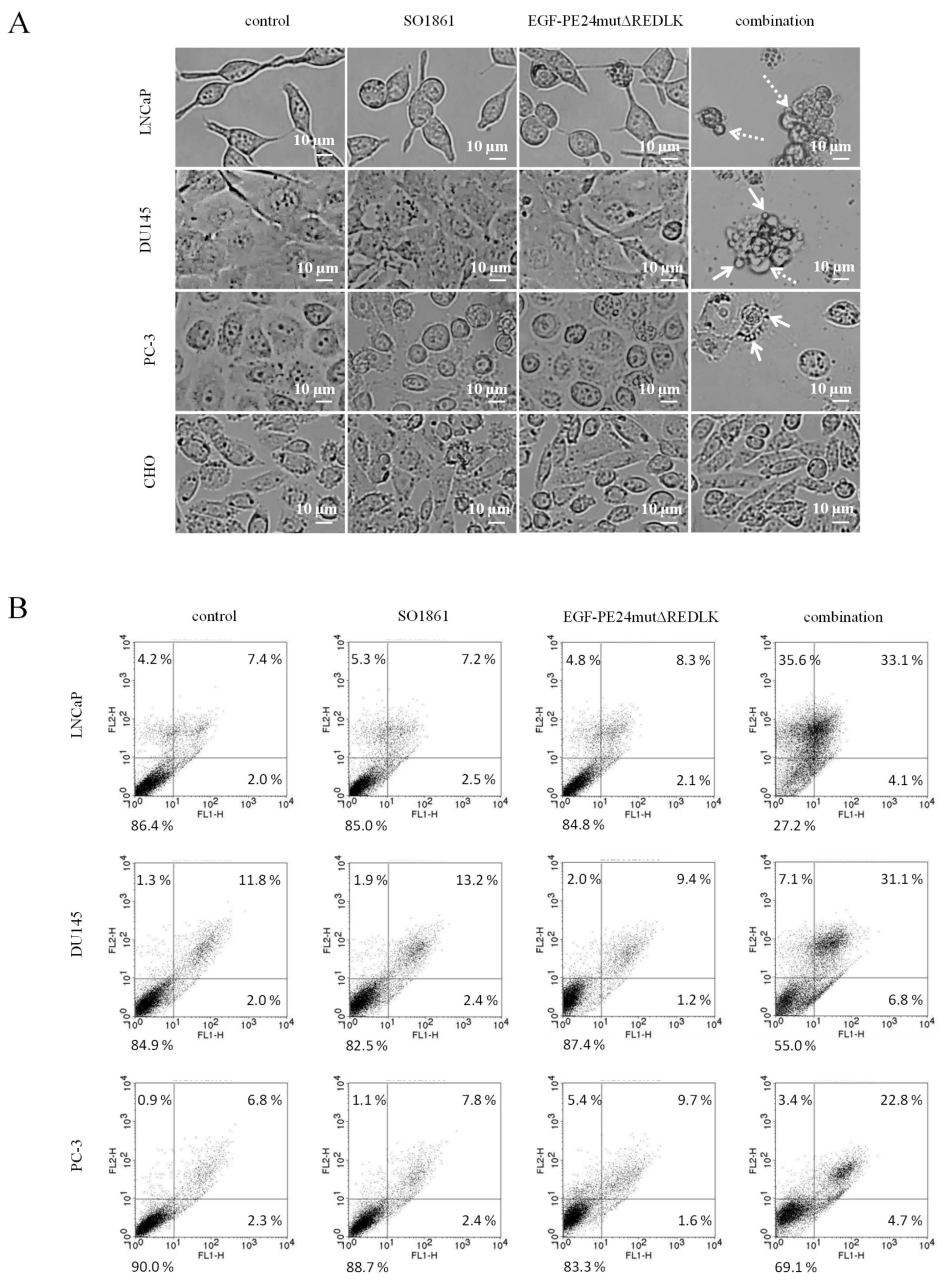
** Induction of apoptosis and necrosis in prostate cancer cells after treatment with EGF-PE24mutΔREDLK and SO1861. (A)** Cell morphology in phase-contrast microscopy at 20× magnification after 48 h or 72 h single or combination treatment with 2.5 nM EGF-PE24mutΔREDLK plus 1.0 µg/ml SO1861. Morphological changes during apoptosis were cell rounding, shrinking, blebbing (dotted arrow) and formation of apoptotic bodies (arrows). **(B)** Cells were stained with AV (FL1-H) and EthD-III (FLH-2) and analyzed by flow cytometry to distinguish between living cells (AV^-^/ EthD-III^-^), early apoptosis (AV^+^/ EthD-III^-^), late apoptosis (AV^+^/ EthD-III^+^) and necrosis (AV^-^/ EthD-III^+^).

Based on these results and on observations that glycosylated triterpenoids promote the endosomal escape of ribosome-inactivating proteins 30, 31, we hypothesized that EGF-PE24mutΔREDLK is released by SO1861 into the cytosol enabling the toxin to inhibit protein biosynthesis and induce apoptosis. Accordingly, we analyzed, if combination treatment led to protein biosynthesis inhibition by performing a puromycin Western Blot. As shown in Fig. 3A, incubation of the prostate cancer cells with EGF-PE24mutΔREDLK and SO1861 resulted in a complete inhibition of protein biosynthesis after 48 or 72 h, respectively. EGFR-negative CHO cell were not affected by this treatment. Inhibition of protein biosynthesis was accompanied by an induction of apoptosis, marked by PARP cleavage and caspase-3 activation (Fig. 3B). Phase-contrast microscopy verified hallmarks of apoptosis after SO1861 enhanced targeted toxin therapy like cell rounding, shrinking, blebbing and formation of apoptotic bodies (Fig. 4A). Annexin V (AV) and Ethidium Homodimer III (EthD-III) staining was done to quantify the percentage of apoptotic and necrotic target cells after treatment with 2.5 nM EGF-PE24mutΔREDLK and 1.0µg/ml SO1861. After 48 h, the percentage of apoptosis (summary of early apoptosis (AV^+^/EthD-III^-^) and late apoptosis (AV^+^/EthD-III^+^)) was between 9.4 % and 10.4 % in untreated LNCaP cells or in LNCaP cells treated with monotherapies. After combinatorial treatment, it increased to 37.2 %. Moreover, percentage of necrotic cells (AV^-^/EthD-III^+^) also increased from basic values between 4.2 and 5.3 % to 33.6 %. Apoptosis was detected in 37.9 % and 27.5 % of the DU145 or PC-3 cells, respectively, after 72 h treatment with EGF-PE24mutΔREDLK and SO1861, while in these two cell lines almost no necrosis was induced (Fig. 4B). Taken together, our analyses revealed that the combination treatment led to complete protein biosynthesis inhibition followed by induction of apoptosis and partially necrosis in the prostate cancer cells.

## Discussion

We developed new anti-EGFR targeted toxins for the treatment of prostate cancer. We used human EGF as binding domain and the de-immunized PE variant PE24mut, which contains domain deletions for a reduced size and mutations of immunodominant epitopes 32. Our targeted toxins are therefore expected to elicit reduced immunogenicity compared to targeted toxins having murine parts in the binding domain, e.g. when they are derived from the chimeric anti-EGFR mAb cetuximab, and having the natural bacterial toxin domain 33, 34. Further studies are needed to clarify if the 34 kDa protein shows higher tumor penetration than larger PE-based targeted toxins having a full IgG antibody or a single chain variable fragment (scFv) instead of the small EGF ligand as binding domains 33, 34. The targeted toxin EGF-PE24mut showed high and specific cytotoxicity in EGFR-expressing prostate cancer cells, which was more than 600 to 3,000-fold cytotoxic than the EGFR inhibitor erlotinib 9. The high potency of targeted toxins can be attributed to their intrinsic enzymatic activity, as only a few toxin molecules are required to catalyze the ADP-ribosylation of many eEF-2 molecules and effectively inhibit protein biosynthesis. In contrast, with competitive inhibitors, such as erlotinib, a stoichiometric one to one binding is necessary for target inhibition.

For enhanced efficacy we decided to combine our targeted toxin with SO1861, which was shown to enhance the endosomal escape of other targeted toxins containing ribosome inactivating proteins, like dianthin or saporin 35-37, but not that of EGF-ETA' (targeted toxin with a toxin domain (aa 276-638) of PE), which was attributed to different intracellular trafficking 38. In a first step, we therefore deleted the C-terminal KDEL-like sequence to generate EGF-PE24mutΔREDLK. As suspected, the cytotoxicity of this targeted toxin variant was markedly reduced by this deletion. Simultaneous application of SO1861, however, led to a 26.6- to 6,966-fold enhanced cytotoxicity in prostate cancer cells, which was proved to be based on the inhibition of protein biosynthesis and induction of apoptosis. The presence of a AV^-^/ EthD-III^+^ population in LNCaP cells after combination treatment can be interpreted as secondary necrosis, an autolytic process of cell disintegration with release of cell components that occurs, when the apoptotic program is completed and when no phagocytic cells are present 39.

Our findings indicate that the targeted toxin was not able to travel via the KDEL receptor mediated pathway, but instead was enriched in the endolysosomes and released by SO1861 into the cytosol to ADP-ribosylate eEF-2 and induce apoptosis. Our results are in line with a publication of Gilabert-Oriol et al., who investigated the mode of action of SO1861 for endolysosomal escape of the toxin dianthin on a molecular level by fluorescence microscopy 36 and a publication of Thakur et al., where a 6,900-fold enhanced cytotoxicity of Sap3-EGF, consisting of EGF and the toxin saporin, was reached after addition of SO1861 in transfected breast cancer cells 40. Besides saponins, photochemical internalization can be used for enhanced endolysosomal release of targeted toxins. Yip and colleagues generated an anti- EGFR targeted toxin consisting of cetuximab as binding domain and saporin from *Saponaria officinalis* as toxin domain. Coincubation of DU145 cells with the targeted toxin and the photosensitizer TPPS2a (meso-tetraphenylporphine disulfonate) led to enhanced cytotoxicity after irradiation with blue light 41.

When testing targeted toxins against EGFR, a correlation between enhanced cytotoxicity and enhanced EGFR expression on different cell lines was found 34. Although all prostate cancer cell lines we tested to have comparable EGFR expression 9, we determined LNCaP cells to be most sensitive to combination treatment. Possible underlying reasons could be different internalization rates, altered intracellular trafficking routes or an increased susceptibility to apoptosis. LNCaP cells represent prostate cancer cells in an advanced, hormone-sensitive stage, whereas DU145 and PC-3 cells originate from hormone-insensitive (castration-resistant) prostate tumors after hormone therapy failure, which are characterized by a high apoptosis resistance 42. Our novel therapeutic approach might therefore preferably be used in hormone-sensitive stages of the disease.

We also observed a 4- to 150-fold cytotoxicity of EGF-PE24mut after coincubation with SO1861. This is in contrast to our experiments with a PE24mut-based anti-PSMA targeted toxin, for which no enhancement of cytotoxicity after addition of SO1861 was observed on the same cell lines 43. Consequently, endosomal release of targeted toxins by enhancers like SO1861 might also be dependent on the kind of binding domain (e.g. antibody or ligand), kind of target antigen, and on the speed, rate, and route of internalization of the targeted toxin. Indeed, the experiments with anti-PSMA antibodies revealed that the internalization of PSMA occurs via clathrin-mediated endocytosis, micropinocytosis and clathrin- and caveolae-independent pathways within a few minutes 44. In contrast, internalization of EGFR after EGF binding mainly occurs via the clathrin-mediated pathway and lasts several hours 45.

The combined application of EGF-PE24mutΔREDLK and SO1861 on prostate cancer cells led to a 16- to 300-fold enhanced cytotoxicity compared to EGF-PE24mut. This provided evidence that it was not only possible to restore the cytotoxicity of the parental targeted toxin, but also to improve it. Figuratively speaking, the synergistic combination allowed the targeted toxin concentration to be reduced to 0.3 to 6.3% of the original dose, which is supposed to reduce undesired effects *in vivo*.

Due to its high and specific cytotoxicity, synergistic treatment with the non-toxic targeted toxin EGF-PE24mutΔREDLK and SO1861 represents a new promising strategy for the future therapy of advanced prostate cancer. Future experiments with patient-derived tumors and in animals with prostate tumor xenografts are necessary to further explore the synergism of both drugs and to find out whether the approach can be transferred into the clinic. With our new treatment option, targeted toxin doses could be effectively reduced and patients could benefit from potent antitumor effects and less treatment boundaries due to severe dose-limiting side effects.

## Figures and Tables

**Table 1:** ** Binding of the targeted toxins EGF-PE24mut and EGF-PE24mutΔREDLK to prostate cancer cells.** Binding was measured by flow cytometry and equilibrium dissociation constants (K_d_) were determined using the software GraphPad Prism 8.01.

	LNCaP	DU145	PC-3	CHO
	K_d_ (nM)	K_d_ (nM)	K_d_ (nM)	K_d_ (nM)
EGF-PE24mut	35.0	36.9	26.6	ND
EGF-PE24mutΔREDLK	23.4	23.2	32.3	ND

Abbreviations: ND, not determinable.

**Table 2:** ** Cytotoxicity of the targeted toxins EGF-PE24mut and EGF-PE24mutΔREDLK alone and in combination with SO1861 in EGFR expressing prostate cancer cells.** CHO cells served as antigen-negative control. IC_50_ values, defined as half-maximal inhibitory concentrations, were determined by WST-1 viability assay and the software GraphPad Prism 8.01.

	LNCaP	DU145	PC-3	CHO
	IC_50_ (nM)	IC_50_ (nM)	IC_50_ (nM)	IC_50_ (nM)
	48 h	72 h	72 h	72 h
EGF-PE24mut	0.9	15.8	1.6	> 20.6
EGF-PE24mut + 1 µg/ml SO1861	0.006	0.3	0.4	> 20.6
-fold enhancement	150	52,7	4	n.d.
EGF-PE24mutΔREDLK	> 20.9	> 20.9	> 20.9	> 20.9
EGF-PE24mutΔREDLK + 1 µg/ml SO1861	0.003	0.8	0.1	> 20.9
-fold enhancement	> 6,966	> 26.2	> 209	n.d.

**Table 3:**
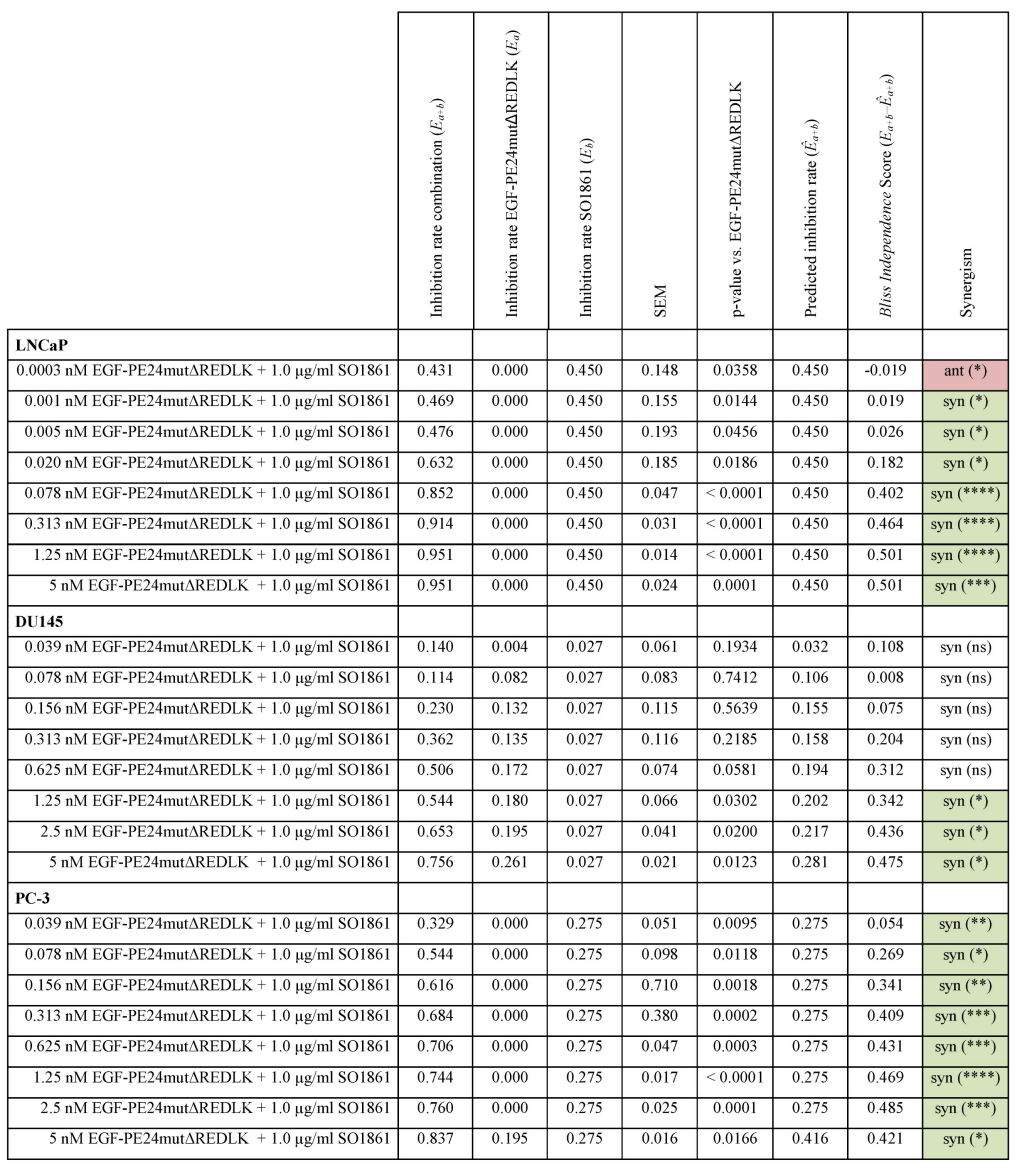
** Statistical analysis and characterization of the SO1861-enhanced targeted toxin therapy on prostate cancer cells.** Statistical analysis according to the *Bliss Independence* score revealed the interaction quality based on the difference between the measured and the predicted inhibition rate. Values > 0 indicate synergism, values < 0 indicate antagonism. The cumulative therapy effect of both individual substances (*Ea + Eb*) minus (*EaEb*) corresponds to the predicted inhibition rate (*Êa+b*). For all targeted toxin concentrations tested between 0.0003 and 5 nM in LNCaP cells and 0.039 and 2.5 nM in PC-3 cells, the predicted inhibition rate is equivalent to the inhibition rate of SO1861 alone, as EGF-PE24mutΔREDLK was not cytotoxic in single application. MW ± SEM was calculated from three independent experiments. P-values were calculated by unpaired *t-*test with Welch's correction.

Abbreviations: *E*, treatment effect; syn, synergistic; ant, antagonistic; ns, not significant; *, p < 0.05; **, p < 0.01; ***, p < 0.001; ****, p < 0.0001.
